# Modified Palygorskite Improves Immunity, Antioxidant Ability, Intestinal Morphology, and Barrier Function in Broiler Chickens Fed Naturally Contaminated Diet with Permitted Feed Concentrations of *Fusarium* Mycotoxins

**DOI:** 10.3390/toxins10110482

**Published:** 2018-11-20

**Authors:** Yefei Cheng, Qiao Xu, Yueping Chen, Yue Su, Chao Wen, Yanmin Zhou

**Affiliations:** College of Animal Science and Technology, Nanjing Agricultural University, Nanjing 210095, China; 2017205020@njau.edu.cn (Y.C.); 2015105058@njau.edu.cn (Q.X.); chenyp0321@163.com (Y.C.); 2015108024@njau.edu.cn (Y.S.); wenage@163.com (C.W.)

**Keywords:** modified palygorskite, *Fusarium* mycotoxins, immunity, oxidative status, intestinal barrier integrity, broiler chickens

## Abstract

This study investigated effects of modified palygorskite (MPal) on immunity, antioxidant capacity, and intestinal barrier integrity in broiler chickens challenged with permitted feed *Fusarium* mycotoxin concentrations. One-day-old chicks were allocated into three treatments with eight replicates. Chickens in three groups were fed a basal diet with normal corn (control), contaminated diet containing moldy corn, with *Fusarium* mycotoxins contents in the diets lower than permitted feed mycotoxin concentrations, and the contaminated diet supplemented with 1 g/kg MPal for 42 days, respectively. Compared with control, moldy corn decreased bursa of Fabricius weight, jejunal secreted immunoglobulin A concentration, ileal superoxide dismutase (SOD) activity, jejunal and ileal villus height (VH) and VH/crypt depth (CD) ratio, and jejunal zonula occludens-1 and mucin 2 mRNA abundances at 42 days as well as ileal VH/CD ratio at 21 days; while they increased jejunal malondialdehyde accumulation at 21 and 42 days, jejunal SOD activity at 21 days, and serum diamine oxidase activity at 42 days, which were almost recovered by MPal. Moreover, dietary MPal upregulated ileal claudin-2 mRNA abundance compared with other two groups. The results indicated that MPal addition exerted protective effects on immunity, oxidative status, and intestinal barrier integrity in chickens challenged with permitted feed *Fusarium* mycotoxins levels.

## 1. Introduction

Mycotoxins, the secondary metabolites produced by fungal species, are the most frequently occurring contaminants in human foods and animal diets. It has been estimated that more than 25% of cereals are contaminated by mycotoxins annually around the world [1]. *Fusarium* mycotoxins—including zearalenone and deoxynivalenol, the most common mycotoxins globally—are produced by *Fusarium* fungi, and more easily generated when cereals and forages are harvested and/or stored at favorable humidity and temperature conditions [2,3]. Undoubtedly, there are always certain levels of *Fusarium* mycotoxins contamination in the crops, and their exposure therefore result in permanent health risk for human beings and farm animals. The cytotoxicity resulting from *Fusarium* mycotoxin contamination has been reported in cell culture experiments [4,5,6]. Moreover, available studies have already demonstrated that higher dosages of *Fusarium* mycotoxins exceeding permitted feed concentrations of mycotoxins could induce impairment on animals including broiler chickens and pigs, as characterized by growth retardation, immunosuppression, oxidative stress, and/or reproductive disorders [7,8,9,10,11,12]. However, those kinds of mycotoxin levels in the animals’ feed are strictly forbidden, and various dosages of *Fusarium* mycotoxins that lower than permitted feed of mycotoxins concentrations are more common in practice. In pigs, Chen et al. have reported that permitted feed concentrations of *Fusarium* mycotoxins can cause significant physiological effects including serum enzymes, inflammatory response, and histopathological changes in the tissues during a six-week study [13]. Meanwhile, it has been demonstrated that zearalenone challenge with permitted feed mycotoxin level could reduce nutrient digestibility, imbalance fecal microflora, and induce oxidative stress in weaned gilts [14]. These findings suggest a possibility that a relative low level of *Fusarium* mycotoxins may induce negative effects on broiler chickens, and more investigations therefore need be conducted to test this hypothesis. The intestinal mucosal barrier consists of a single layer of tightly-arranged epithelial cells that largely joined together by junctional complexes, including tight junctions [15]. This physical selective barrier represents the first barrier against food contaminants and natural toxins, and simultaneously regulates small molecule transport and intestinal epithelial cells permeability [15]. Published papers have illustrated that *Fusarium* mycotoxin contamination with high dosages could damage intestinal morphology and barrier function in broiler chickens and pigs [7,11,16,17,18]. Moreover, similar findings on pigs resulting from low levels of *Fusarium* mycotoxin contamination were also observed as previously reported [19,20]. However, little information is available in terms of low levels of *Fusarium* mycotoxins challenge on this barrier function in broiler chickens.

In animal production, various strategies have been developed, such as physical, chemical, microbiological, and nutritional methods, to eliminate or attenuate the deleterious effects of mycotoxins including *Fusarium* mycotoxins in contaminated feeds [21]. The supplementation of binding adsorbent in the diets is so far a more practical approach, which can adsorb mycotoxin molecules to preclude their absorption from the intestine, thereby ameliorating their toxic effects on animals [22]. The most commonly used adsorptive materials are clays, for example, montmorillonite, zeolite, and kaolinite [21]. However, those clays exerted poor adsorption capacity to nonpolar and/or less polar mycotoxin molecules, such as *Fusarium* mycotoxins, due to their hydrophilic negatively charged surfaces [21,23,24,25]. Previous in vitro and in vivo studies have illustrated that those modified clays can increase their surface hydrophobicity, exhibit a stronger adsorptive ability to nonpolar mycotoxin [26,27,28], and attenuate the detrimental effects of those mycotoxins on livestock [17,27,29,30].

Palygorskite (Pal) is a naturally available hydrated magnesium-rich silicate clay mineral with nanorod-like crystal morphology and nano-channels. It has been demonstrated that dietary Pal addition could efficiently adsorb aflatoxin mycotoxin [31], and alleviate aflatoxin-induced damages in pigs [32,33]. Additionally, the supplementation of Pal could improve antioxidant capacity, immune response, and/or intestinal barrier integrity of animals including broiler chickens [34], pigs [35], and laying hens [36]. Nevertheless, similar to other clays, Pal also had weak affinity with nonpolar mycotoxin molecules. It is accordingly necessary that Pal needs to be modified (modified Pal, MPal) to improve its adsorption capacity of nonpolar mycotoxins. Recent studies in our lab have shown that Pal modified with an organic cation surfactant could improve intestinal oxidative status, immunity, and/or barrier integrity in laying hens and broiler chickens fed with a normal diet [37,38]. Moreover, this MPal exerted a better adsorptive capacity to zearalenone [39] and also efficiently reduced hepatic zearalenone residue as well as relieved hepatic oxidative damage in broiler chickens given a diet contaminated with zearalenone [40]. In consideration of these aforementioned findings, we hypothesized that a relatively low level of *Fusarium* mycotoxin contamination may induce detrimental consequences on intestines in broiler chickens, and the supplementation of this MPal into the contaminated feed would exert protective effects. The present study was therefore conducted to investigate effects of contaminated diet with permitted feed levels of *Fusarium* mycotoxins that derived from naturally moldy corn on immunity, and intestinal antioxidant ability, morphology, and barrier function of broiler chickens, and also verify the efficacy of MPal supplementation on broiler chickens fed the contaminated diet.

## 2. Results

### 2.1. Relative Immune Organ Weight

Compared with the control group (Table 1), broiler chickens receiving *Fusarium* mycotoxin-contaminated diet exhibited a decrease in bursa of Fabricius relative weight at 42 days (*p* < 0.05), whereas this effect was not observed at 21 days (*p* > 0.05). MPal supplementation increased relative bursa of Fabricius weight of broiler chickens at 42 days compared with the mycotoxins group (*p* < 0.05). However, relative thymus and spleen weights were similar among treatments (*p* > 0.05).

### 2.2. Immunoglobulin Concentration in the Intestine

Secretory immunoglobulin A (SIgA) concentration in the jejunal and ileal mucosa of broiler chickens at 42 days in the mycotoxin group was lower than that in the control group (Table 2, *p* < 0.05), and it was recovered to a normal level by MPal addition (*p* < 0.05). However, neither feeding moldy corn nor MPal inclusion affected IgG and IgM contents in the intestinal mucosa of broiler chickens (*p* > 0.05).

### 2.3. Intestinal Antioxidant Status

*Fusarium* mycotoxin-contaminated diet increased jejunal malondialdehyde (MDA) concentration at both 21 and 42 days and jejunal superoxide dismutase (SOD) activity at 42 days (Table 3), whereas decreased ileal SOD activity at 42 days when compared with those of broiler chickens fed the normal corn (*p* < 0.05). In contrast, MPal supplementation reduced jejunal MDA concentration at both 21 and 42 days and jejunal SOD activity at 42 days but increased ileal SOD activity at 42 days compared with the mycotoxins group (*p* < 0.05), with the values of SOD activity and MDA content in the jejunal mucosa being similar to the control group (*p* > 0.05), and ileal SOD activity still being higher than the control group (*p* < 0.05). Treatments, however, did not alter intestinal antioxidant capacity of broiler chickens at 21 days (*p* > 0.05).

### 2.4. Serum Diamine Oxidase (DAO) Activity and Intestinal Morphology

Broiler chickens receiving moldy corn exhibited an increase in serum DAO activity at 42 days, whereas decreases in VH and VH: CD ratio in the jejunum and ileum at 42 days as well as ileal VH: CD ratio at 21 days compared with the control group (*p* < 0.05, Table 4). In contrast, MPal inclusion decreased serum DAO activity and attenuated disruption of intestinal morphology of broiler chickens ingesting moldy corn (*p* < 0.05).

### 2.5. Gene Expressions Related to Intestinal Barrier Function

Treatments did not affect intestinal mucosal mRNA expression related to barrier function at 21 days (Table 5, *p* > 0.05). Compared with the control group, *Fusarium* mycotoxin contamination reduced mRNA abundances of mucin 2 (MUC2) and zonula occludens-1 (ZO-1) in the jejunual mucosa at 42 days (*p* < 0.05). The supplementation of MPal increased jejunual ZO-1 gene expression at 42 days compared with the mycotoxin group (*p* < 0.05) to a level comparable with the control group (*p* < 0.05). Additionally, MPal inclusion also upregulated ileal claudin-2 (CLDN2) expression abundance when compared with the other two groups (*p* < 0.05).

## 3. Discussion

Relative organ weight could reflect the growth and development of organ at some extent. The effects of *Fusarium* mycotoxins on relative immune organ weight have already been investigated, the results, however, are contradictory among those researches [7,41,42,43]. In the present study, decreased relative weight of bursa of Fabricius in broiler chickens fed the *Fusarium* mycotoxins-contaminated diet was observed, suggesting that *Fusarium* mycotoxins with permitted feed concentrations of mycotoxins could suppress immune function of broiler chickens. Consistently, previous studies have illustrated that *Fusarium* mycotoxin contamination could disrupt immune organ development (swell or atrophy) in animals [7,41,42,44]. However, it has also been reported that *Fusarium* mycotoxins did not affect immune organ index in animals in published papers [43,45,46]. The discrepancy may be related to the dosage, duration, and type of mycotoxin, species, and management. The primary function of SIgA is to limit the access of pathogenic bacteria and mucosal antigens to the mucosal barrier [47]. The finding of this experiment showed that the feeding of moldy corn contaminated with *Fusarium* mycotoxins decreased SIgA concentration in the jejunal and ileal mucosa of broiler chickens at 42 days. Reduced intestinal SIgA content, coupled with simultaneously decreased relative bursa of Fabricius weight in this study, further indicated that the permitted feed of *Fusarium* mycotoxins could induce immunosuppression in broiler chickens. This result was consistent with some reports, in which *Fusarium* mycotoxins decreased IgM, IgG, and/or SIgA (IgA) concentrations in animals [8,48]. In broiler chickens, Chen et al. have reported that dietary Pal supplementation could improve immunity via enhancing intestinal IgM and SIgA contents [34]. Similarly, improved immunity of broiler chickens was observed by appropriate levels of MPal addition in a recent investigation, as evidenced by increased IgM, IgG, and SIgA contents in the intestinal mucosa at 42 days [37]. Moreover, it has been proven that a modified clay (montmorillonite) could elevate serum IgG concentration in laying hens [49]. However, few studies are available about the effects of aforementioned MPal on immunity in broiler chickens fed moldy contaminated with *Fusarium* mycotoxins. In the current study, we observed that dietary MPal supplementation increased relative bursa of Fabricius weight and SIgA concentration in the jejunal and ileal mucosa at 42 days, and it was in agreement with the results by Yin et al. [48] and Weaver et al. [50]. Researches from our lab have illustrated that this MPal exerted better adsorption capacity to zearalenone [39], and improved immunity, intestinal antioxidant status, as well as barrier integrity of animals [37,38], which would, partially and eventually, result in better immune function of broiler chickens challenged with permitted feed concentrations of *Fusarium* mycotoxins in the present study.

Appropriate levels of free radicals by living organisms are necessary during normal cellular metabolism, its overproduction exceeding capacity of antioxidant defense system, however, could result in oxidative damage. Lipid peroxidation is a process where carbon-carbon double bonds are attacked by free radicals, and MDA is the end product of lipid peroxidation, and its accumulation could therefore be used as a reliable index for lipid peroxidation [51]. SOD is one of the most important lines of antioxidant enzyme and plays a vital role in scavenging excessive free radicals [52]. Previous in vivo studies have proven that high levels of *Fusarium* mycotoxin contamination could accelerate lipid oxidation and disrupt antioxidant enzymes in the tissues, eventually resulting in oxidative damage [9,10,11]. Moreover, piglets challenged with low dosages of zearalenone exhibited increased MDA content whereas decreased SOD activity in the liver (or serum) [14,53]. Consistently, the finding of this work showed that the feeding of moldy corn increased jejunal MDA concentration at both 21 and 42 days and jejunal SOD activity at 42 days, whereas decreased ileal SOD activity at 42 days, implying that the relative low level of *Fusarium* mycotoxin contamination would result in intestinal oxidative damage. Interestingly, SOD activity was increased in the jejunal mucosa whereas it was decreased in the ileal mucosa resulting from *Fusarium* mycotoxins, we speculated it might be related to the different antioxidant capacities of different intestinal segments, and mycotoxins may activate jejunal antioxidant ability while suppressing ileal antioxidant capacity. In broiler chickens, Chen et al. have reported that Pal supplementation into diet could increase intestinal SOD activity [34]. Moreover, our laboratory members have currently demonstrated that MPal aforementioned could improve intestinal antioxidant status in broiler chickens and laying hens, as evidenced by decreased MDA concentration while increasing antioxidant enzymes including SOD and total-antioxidant capacity [37,38]. In the present study, we observed that this MPal reduced jejunal MDA content at both 21 and 42 days and jejunal SOD activity at 42 days, whereas increased ileal SOD activity at 42 days when compared with the mycotoxins group. These results were in accordance with the findings by Zhang et al. and Jiang et al., who have demonstrated that other modified clays could reduce MDA concentration whereas increased antioxidant enzymes including SOD and glutathione peroxidase activities in the tissues of animals challenged with *Fusarium* mycotoxins [27,53]. The improved intestinal antioxidant capacity of broiler chickens fed permitted feed levels of *Fusarium* mycotoxins resulting from MPal supplementation in the present study may attribute to its better adsorption ability to *Fusarium* mycotoxins [39]. Additionally, the promotion of MPal on intestinal barrier integrity, immunity, and antioxidant status in animals would also account for the better antioxidant ability of broiler chickens administered moldy corn [37,38].

The normal microarchitecture of small intestine is very crucial in maintaining nutrient absorption and resistance to harmful substrates, and therefore plays a vital role in growth and development for individual. VH and CD serve as criteria that reflect gross intestinal morphology. Available studies have illustrated that *Fusarium* mycotoxins could induce progressive deterioration on intestinal histology of animals, resulting in a shorter VH and a lower VH: CD ratio, and/or a deeper CD in the intestine [11,19,44,54]. Similarly, decreased VH and VH: CD ratio in the jejunum and ileum at 42 days as well as reduced VH: CD ratio in the ileum at 21 days was observed in the mycotoxins group when compared with the control group in the present study. It has been reported that mycotoxin could exert inhibitory effects on eukaryotic cells via inhibiting cell division and synthesis of RNA, DNA, as well as protein; stimulating ribotoxic stress response and activating mitogen-activated protein kinases, important signal proteins that regulate cellular proliferation, differentiation, and apoptosis [55]. These adverse consequences may eventually result in intestinal villus atrophy. A more efficient way to evaluate the effect of dietary manipulation on gastrointestinal health is the measurement of intestinal morphology. Several studies from our lab have proven that dietary supplementation with either Pal or aforementioned MPal could increase VH and VH: CD ratio, and/or decrease CD in broiler chickens [34,36,38]. In the current study, MPal supplementation increased jejunal and ileal VH and VH: CD ratio at 42 days as well as ileal VH: CD ratio at 21 days as compared with the mycotoxins group. Those results indicated that dietary MPal inclusion is an efficient method in ameliorating compromised intestinal morphology of broiler chickens fed the low level of *Fusarium* mycotoxins-contaminated diet.

DAO is mainly localized in the small intestinal mucosa and reflects intestinal integrity and maturity [56]. Intestinal mucosal damage can induce DAO leakage from intestinal mucosa into the circulation [57]. Therefore, increased serum DAO activity is commonly associated with intestinal permeability and mucosal injury. In the present study, the permitted feed concentrations of *Fusarium* mycotoxins increased serum DAO activity in broiler chickens at 42 days. Similarly, Wu et al. have reported that piglets challenged with deoxynivalenol exhibited a higher serum DAO activity [58]. MUC2 is a main component of gut mucus that covers the surface of intestinal mucosa, and can repair intestinal mucosal injury induced by harmful factors [59]. Tight junctions are the crucial components of the intestinal mucosal barrier. They mainly consist of peripheral membrane protein ZO-1 and the transmembrane protein OCLN and claudins [60]. Herein, the downregulation of MUC2, ZO-1, OCLN, and claudins mRNA expressions levels would be harmful to intestinal structure and barrier function. In vitro studies have already illustrated that *Fusarium* mycotoxins could downregulate ZO-1, OCLN, and/or claudin protein expressions in several cell lines [61,62,63]. In vivo studies have further demonstrated aforementioned findings as well [7,11,16,54]. Consistently, this research showed that broiler chickens fed moldy corn exhibited decreases in mRNA abundances of MUC2 and ZO-1 in the jejunual mucosa at 42 days. These findings, together with compromised intestinal morphology, suggested that with *Fusarium* mycotoxin contamination, lowering permitted feed concentrations of mycotoxins could impair intestinal barrier integrity of broiler chickens. It has been reported that dietary Pal supplementation could improve intestinal integrity of broiler chickens, as evidenced by decreased serum DAO activity whereas upregulated genes expressions of intestinal mucosal MUC2 and ZO-1. Meanwhile, a better intestinal integrity of weaned piglets resulting from Pal addition was also observed by Zhang et al. [35]. Moreover, recent studies from our lab have proved that abovementioned MPal can improve intestinal barrier function of broiler chickens and laying hens, as illustrated by reduced serum DAO activity and/or increased genes expressions related to intestinal barrier integrity. However, no research was conducted to investigate whether this kind of MPal could exert beneficial consequences on intestinal barrier function of broiler chickens ingesting moldy corn contaminated with *Fusarium* mycotoxins. In the current study, dietary MPal supplementation increased jejunual mucosal ZO-1 gene and ileal mucosal CLDN2 gene expressions levels, whereas decreased serum DAO activity at 42 days compared with the mycotoxins group, suggesting that dietary MPal supplementation exerted protective effects on damaged intestinal barrier integrity resulting from the permitted feed concentrations of *Fusarium* mycotoxins. The better intestinal barrier integrity of broiler chickens fed moldy by MPal supplementation in this study, on the one hand, attributed to the improved intestinal antioxidant status and immunity simultaneously, on the other hand, result from the improvement of Pal and MPal on intestinal morphology and barrier function in animals [34,37,38].

## 4. Conclusions

The results of our study demonstrated that the permitted feed concentrations of *Fusarium* mycotoxins derived from moldy corn could compromise immune function and intestinal antioxidant capacity as well as barrier integrity in broiler chickens. The supplementation of dietary MPal was efficient in ameliorating those adverse consequences on broiler chickens challenged with the low level of *Fusarium* mycotoxin contamination.

## 5. Materials and Methods

All procedures in the present study were performed in full compliance with recommendation of Nanjing Agricultural University Institutional Animal Care and Use Committee (certification No. SYXK (Su) 2011-0036, 11 August 2015).

### 5.1. Materials

MPal used in this study was the same as that in our recent trials [38]. Additionally, a variety of moldy corn samples were collected from different places for subsequent measurement of mycotoxin contents including aflatoxin B_1_, deoxynivalenol, and zearalenone. The moldy corn samples with relative high concentrations of zearalenone and/or deoxynivalenol were selected, and then used to replace normal corn in the basal diet for *Fusarium* mycotoxin-contaminated diet preparation, with the *Fusarium* mycotoxin concentrations in the contaminated diets being permitted by Chinese Ministry of Agriculture.

### 5.2. Animals, Diets, and Experimental Design

A total of 192 one-day-old female Arbor Acres plus broiler chicks with similar initial weights were randomly allocated to three treatment groups, and each group consisted of eight replicates (cages) with eight birds per cage. Broiler chickens in the three groups were fed a basal diet with normal corn (control group), contaminated diet with permitted feed concentrations of *Fusarium* mycotoxins (mycotoxins group), and the *Fusarium* mycotoxin-contaminated diet supplemented with 1 g/kg MPal (MPal group) for 42 days, respectively. The basal diet is formulated according to the recommendation by NRC [64], and the contaminated diets were prepared by using the naturally *Fusarium* mycotoxin-contaminated corn to equally replace normal corn in the control group. Representative samples of diets among three groups were collected for nutrients and mycotoxin contents analysis before the feeding trail. The components and nutrient levels of diets are shown in Table 6. The aflatoxin B_1_, deoxynivalenol, and zearalenone concentrations of diets were determined by enzyme-linked immunosorbent assay (ELISA) according to the manufacturer’s instructions (R-Bio-pharm, Darmatadt, Germany), and also presented in Table 6. Chickens were housed in a temperature-controlled room, and had free access to clean water and mash feed in the three-layer-wired cages (120 × 60 × 50 cm; 0.09 m^2^ per bird), and continuous light in the housing room was provided. The temperature in the housing room was similar to that as reported in our previous studies [65].

### 5.3. Sample Collection

At 21 and 42 days of age, one broiler from each cage was selected (close to average body weight of the corresponding cage) and weighed. Blood sample was obtained from jugular vein, and serum was separated and stored at −20 °C prior to analysis after a centrifugation at 3000× *g* for 15 min at 4 °C. Then, broiler chickens were euthanized by cervical dislocation and necropsied immediately. After that, the immune organs including thymus, spleen, and bursa of Fabricius were quickly harvested and weighed for calculating relative immune organ weight, which was expressed as g/kg live body weight. Additionally, the whole gastrointestinal tract was also rapidly removed, and the segments of mid-jejunum and mid-ileum were excised (about 2 cm) and flushed gently with ice-cold phosphate-buffered saline to remove the contents, which were thereafter placed in 10% neutral-buffered for morphology measurement. The remaining jejunum and ileum were subsequently opened longitudinally and the contents were flushed with ice-cold and sterile phosphate-buffered saline. The jejunal and ileal mucosa was then scratched carefully using a sterile glass microscope slide and collected into sterile frozen tubes, which was snapped in liquid nitrogen, and then frozen at −80 °C until analysis.

### 5.4. Determination of Serum DAO Activity

The DAO activity in the serum was assayed using the available kit (Nanjing Jiancheng Institute of Bioengineering, Nanjing, China) according to the method described by Hosada et al. [66]. Briefly, serum sample (0.5 mL) was added into reagent containing 3.0 mL of PBS (0.2 mol/L, pH 7.2), 0.1 mL of *o*-dianisidine-methanol solution (5 g/L odianisidine inmethanol), 0.1 mL of horseradish peroxidase solution (0.04 g/L), and 0.1 mL of substrate solution, and incubated for 30 min at 37 °C, which was thereafter measured spectrophotometrically to calculate serum DAO activity.

### 5.5. Measurement of Intestinal Mucosal Parameters

About 0.3 g of each jejunal and ileal mucosa sample was homogenized at a ratio of 1:9 (weight/volume) with ice-cold 154 mmol/L sterile sodium chloride solution employing a PRO-PK-02200D homogenizer (Pro Scientific, Inc., Monroe, CT, USA). Homogenate was centrifuged at 3500× *g* for 10 min at 4 °C to acquire supernatant, and it was immediately frozen at −20 °C for further analysis. The concentrations of SIgA, IgM, and IgG in the jejunal and ileal mucosa were determined using chicken-specific SIgA, IgG, and IgM ELISA quantitation kits according to the corresponding protocols (Nanjing Jiancheng Bioengineering Institute, Nanjing, China). SOD activity and MDA accumulation were measured using commercial kits purchased from Nanjing Jiancheng Institute of Bioengineering (Nanjing, China). MDA content was measured following the thiobarbituric acid method described by Placer et al. [67]. SOD activity was measured by xanthine oxidase method [68]. One unit of SOD activity was defined as the amount of enzyme per milligram protein of muscle that would produce 50% inhibition of the rate of nitrite production at 37 °C. The results were normalized against total protein concentration in each sample for inter-sample comparison. Total protein content of each mucosal sample was determined by a Coomassie brilliant blue protein assay kit (Nanjing Jiancheng Institute of Bioengineering, Nanjing, China) using bovine serum albumin as the standard.

### 5.6. Intestinal Morphology Examination

The harvested segments of jejunum and ileum were dehydrated, cleared, and embedded in paraffin, respectively, after a 24-h fixation in buffered formalin. They were then cut into serial sections at 5-μm depth for subsequent stain with hematoxylin and eosin. VH and CD were determined using a light microscope equipped with a computer-assisted morphometric system (Nikon Corporation, Tokyo, Japan). Values were means from 10 samples of villi well oriented from the tip to the crypt mouth and 10 associated crypts from the crypt mouth to the base. The aforementioned procedures followed the description by Hu et al. [69].

### 5.7. Messenger RNA Quantification

Total RNA from jejunal and ileal mucosa was isolated using Trizol reagent according to the instructions of manufacturer (TaKaRa Biotechnology Co. Ltd., Dalian, China). The final concentration and purity of RNA was quantified using a spectrophotometer (NanoDrop 2000c, Thermo Scientific, USA) from OD260/280 readings obtaining ratios between 1.8 and 2.1. RNA samples were then diluted with diethyl pyrocarbonate-treated water (Biosharp) to a final concentration of 0.5 μg/μL. After that, 1 μg of total RNA was reversed immediately following the RNA isolation using the Perfect Real Time SYBR Prime Script RT Master Mix reagent kit (TaKaRa Biotechnology Co., Ltd., Dalian, China). The primer sequences were obtained from our recent study [69], and synthesized by Invitrogen Biotechnology Co., Ltd. (Shanghai, China), which are listed in Table 7. The cDNA samples were amplified with the SYBR Premix Ex TaqII Tli RNaseH Plus kit based on an ABI7500 Real-time PCR system (Applied Biosystems, Grand Island, NY, USA). Detailed procedures of real-time quantitative PCR were performed following the descriptions by our recent study [70]. Each sample was measured in triplicate, and gene expression was calculated relative to β-actin using the 2*^−^*^ΔΔCT^ method [71].

### 5.8. Statistical Analysis

Statistical analyses of data were performed using SPSS 19.0 for Windows statistical software package (SPSS Inc., Chicago, IL, USA, 2010). Replicate was defined as an experimental unit for the trial. Data were tested by one-way analysis of variance (ANOVA) followed by Tukey’s multiple comparison tests, which were considered significant when *p* < 0.05. Results are presented as means and standard error of means (SEM).

## Figures and Tables

**Table 1:** Effect of MPal supplementation on relative immune organ weight in broiler chickens fed the contaminated diet with permitted feed concentrations of *Fusarium* mycotoxins (g/kg).

Items ^1^	Control Group	Mycotoxins Group	MPal Group	SEM	*p* Value
**21 Days**					
**Thymus**	1.03	1.02	1.00	0.04	0.958
**Spleen**	2.78	2.32	2.64	0.16	0.518
**Bursa of Fabricius**	1.99	2.50	2.28	0.10	0.112
**42 Days**					
**Thymus**	1.31	1.32	1.23	0.06	0.796
**Spleen**	2.42	1.87	1.90	0.12	0.099
**Bursa of Fabricius**	1.74 ^a^	1.15 ^b^	1.70 ^a^	0.09	0.002

^1^ Control group, Mycotoxins group, and MPal group, broiler chickens were given the basal diet, and the *Fusarium* mycotoxins-contaminated diets supplemented with either 0 or 1 g/kg MPal, respectively. SEM, total standard error of means (*n* = 8). ^a,b^ Means within a row with different superscripts are different at *p* < 0.05.

**Table 2:** Effect of MPal supplementation on intestinal immunoglobulin concentration in broiler chickens fed the contaminated diet with permitted feed concentrations of *Fusarium* mycotoxins (μg/mg protein).

Items ^1,2^	Control Group	Mycotoxins Group	MPal Group	SEM	*p* Value
**21 Days**					
**Jejunum**					
**SIgA**	0.78	0.82	0.83	0.02	0.663
**IgG**	11.14	11.10	10.80	0.36	0.928
**IgM**	0.92	0.92	0.88	0.03	0.863
**Ileum**					
**SIgA**	1.07	1.15	1.04	0.03	0.345
**IgG**	14.34	16.61	15.95	0.54	0.225
**IgM**	1.16	1.33	1.27	0.04	0.239
**42 Days**					
**Jejunum**					
**SIgA**	0.87 ^a^	0.60 ^b^	0.77 ^a^	0.03	0.002
**IgG**	11.06	10.28	10.71	0.36	0.714
**IgM**	0.90	0.89	0.87	0.03	0.925
**Ileum**					
**SIgA**	0.83 ^a^	0.67 ^b^	0.93 ^a^	0.04	0.006
**IgG**	10.03	11.34	11.08	0.41	0.403
**IgM**	0.79	0.88	0.86	0.03	0.440

^1^ SIgA, secretory immunoglobulin A; IgG, immunoglobulin G; IgM, immunoglobulin M. ^2^ Control group, Mycotoxins group, and MPal group, broiler chickens were given the basal diet, and the *Fusarium* mycotoxins-contaminated diets supplemented with either 0 or 1 g/kg MPal, respectively. SEM, total standard error of means (*n* = 8). ^a,b^ Means within a row with different superscripts are different at *p* < 0.05.

**Table 3:** Effect of MPal supplementation on intestinal antioxidant capacity in broiler chickens fed the contaminated diet with permitted feed concentrations of *Fusarium* mycotoxins.

Items ^1,2^	Control Group	Mycotoxins Group	MPal Group	SEM	*p* Value
**21 Days**					
**Jejunum**					
**SOD (U/mg Protein)**	220.98	235.27	219.49	4.09	0.213
**MDA (nmol/mg Protein)**	0.97 ^b^	1.41 ^a^	1.11 ^b^	0.06	0.004
**Ileum**					
**SOD (U/mg Protein)**	188.33	182.80	193.03	5.06	0.699
**MDA (nmol/mg Protein)**	0.98	0.97	0.79	0.05	0.236
**42 Days**					
**Jejunum**					
**SOD (U/mg Protein)**	205.27 ^b^	244.47 ^a^	221.6 ^b^	5.41	0.007
**MDA (nmol/mg Protein)**	0.70 ^b^	1.05 ^a^	0.73 ^b^	0.05	<0.001
**Ileum**					
**SOD (U/mg Protein)**	162.14 ^b^	142.38 ^c^	174.73 ^a^	3.41	<0.001
**MDA (nmol/mg Protein)**	0.70	0.79	0.65	0.04	0.400

^1^ SOD, superoxide dismutase; MDA, malondialdehyde. ^2^ Control group, Mycotoxins group, and MPal group, broiler chickens were given the basal diet, and the *Fusarium* mycotoxin-contaminated diets supplemented with either 0 or 1 g/kg MPal, respectively. SEM, total standard error of means (*n* = 8). ^a,b,^^c^ Means within a row with different superscripts are different at *p* < 0.05.

**Table 4:** Effect of MPal supplementation on serum diamine oxidase activity and intestinal morphology in broiler chickens fed the contaminated diet with permitted feed concentrations of *Fusarium* mycotoxins.

Items ^1,2^	Control Group	Mycotoxins Group	MPal Group	SEM	*p* Value
**21 Days**					
**DAO (U/mL)**	11.42	15.80	14.80	1.41	0.419
**Jejunum**					
**VH (μm)**	1191.83	1121.66	1142.70	15.46	0.166
**CD (μm)**	143.76	142.29	141.23	1.50	0.786
**VH: CD**	8.35	7.87	8.13	0.09	0.093
**Ileum**					
**VH (μm)**	971.48	919.05	941.43	14.06	0.330
**CD (μm)**	145.39	153.32	146.75	1.64	0.103
**VH: CD**	6.71 ^a^	6.02 ^b^	6.44 ^a^	0.10	0.004
**42 Days**					
**DAO (U/mL)**	25.58 ^b^	33.04 ^a^	11.32 ^c^	2.77	<0.001
**Jejunum**					
**VH (μm)**	1776.08 ^a^	1459.04 ^b^	1751.13 ^a^	41.76	<0.001
**CD (μm)**	229.72	234.47	234.64	2.16	0.600
**VH: CD**	7.80 ^a^	6.27 ^b^	7.51 ^a^	0.18	<0.001
**Ileum**					
**VH (μm)**	1256.33 ^a^	1015.01 ^b^	1202.71 ^a^	34.03	<0.001
**CD (μm)**	197.45	190.39	194.16	2.63	0.577
**VH: CD**	6.41 ^a^	5.36 ^b^	6.23 ^a^	0.15	0.001

^1^ DAO, diamine oxidase; VH, villus height; CD, crypt depth; VH: CD, villus height/crypt depth. ^2^ Control group, Mycotoxins group, and MPal group, broiler chickens were given the basal diet, and the *Fusarium* mycotoxin-contaminated diets supplemented with either 0 or 1 g/kg MPal, respectively. SEM, total standard error of means (*n* = 8). ^a,b,c^ Means within a row with different superscripts are different at *p* < 0.05.

**Table 5:** Effect of MPal supplementation on intestinal mucosal gene expressions in broiler chickens fed the contaminated diet with permitted feed concentrations of *Fusarium* mycotoxins.

Items ^1,2^	Control Group	Mycotoxins Group	MPal Group	SEM	*p* Value
**21 Days**					
**Jejunum**					
**MUC2**	1.00	0.95	1.22	0.13	0.699
**ZO-1**	1.00	1.20	1.07	0.11	0.789
**OCLN**	1.00	1.02	1.08	0.12	0.966
**CLDN2**	1.00	0.89	1.21	0.12	0.585
**CLDN 3**	1.00	0.93	0.95	0.06	0.875
**Ileum**					
**MUC2**	1.00	0.79	1.11	0.10	0.443
**ZO-1**	1.00	0.90	0.88	0.06	0.749
**OCLN**	1.00	0.67	0.92	0.09	0.270
**CLDN2**	1.00	1.11	1.03	0.11	0.921
**CLDN 3**	1.00	0.92	0.93	0.09	0.919
**42 days**					
**Jejunum**					
**MUC2**	1.00 ^a^	0.60 ^b^	0.84 ^a,b^	0.07	0.046
**ZO-1**	1.00 ^a^	0.62 ^b^	0.89 ^a^	0.06	0.011
**OCLN**	1.00	1.04	1.41	0.12	0.309
**CLDN2**	1.00	0.92	1.10	0.11	0.798
**CLDN 3**	1.00	1.03	1.02	0.09	0.991
**Ileum**					
**MUC2**	1.00	0.84	0.98	0.09	0.792
**ZO-1**	1.00	0.96	0.96	0.11	0.983
**OCLN**	1.00	0.92	1.04	0.12	0.917
**CLDN2**	1.00 ^b^	0.97 ^b^	1.54 ^a^	0.11	0.047
**CLDN 3**	1.00	0.86	0.82	0.07	0.525

^1^ MUC2, mucin 2; ZO-1, zonula occludens-1; OCLN, occludin; CLDN2, claudin-2; CLDN3, claudin-3. ^2^ Control group, Mycotoxins group, and MPal group, broiler chickens were given the basal diet, and the *Fusarium* mycotoxin-contaminated diets supplemented with either 0 or 1 g/kg MPal, respectively. SEM, total standard error of means (*n* = 8). ^a,b^ Means within a row with different superscripts are different at *p* < 0.05.

**Table 6:** Compositions, nutrient levels, and mycotoxin concentrations of diets (%, as fed basis unless otherwise stated).

Items ^1,2^	1–21 Days			22–42 Days		
	Control Group	Mycotoxins Group	MPal Group	Control Group	Mycotoxins Group	MPal Group
**Ingredients**						
**Normal Corn**	57.00	0.00	0.00	62.00	0.00	0.00
**Moldy Corn**	0.00	57.00	57.00	0.00	62.00	62.00
**Soybean Meal**	32.60	32.60	32.60	28.00	28.00	28.00
**Corn Gluten Meal**	3.00	3.00	3.00	2.00	2.00	2.00
**Soybean Oil**	3.00	3.00	3.00	4.00	4.00	4.00
**Limestone**	1.23	1.23	1.23	1.30	1.30	1.30
**Dicalcium Phosphate**	2.00	2.00	2.00	1.60	1.60	1.60
**L-Lysine·HCl**	0.32	0.32	0.32	0.31	0.31	0.31
**DL-Methionine**	0.15	0.15	0.15	0.11	0.11	0.11
**Sodium Chloride**	0.30	0.30	0.30	0.30	0.30	0.30
**Premix**	0.40	0.40	0.40	0.38	0.38	0.38
**Calculated Nutrient Levels**						
**AME (MJ/kg)**	12.56	12.56	12.56	12.98	12.98	12.98
**Crude Protein**	21.55	21.55	21.55	19.33	19.33	19.33
**Lysine**	1.22	1.22	1.22	1.10	1.10	1.10
**Methionine**	0.50	0.50	0.50	0.43	0.43	0.43
**Calcium**	1.01	1.01	1.01	0.93	0.93	0.93
**Available Phosphorus**	0.46	0.46	0.46	0.39	0.39	0.39
**Methionine + Cystine**	0.86	0.86	0.86	0.76	0.76	0.76
**Analyzed Nutrient Levels**						
**Gross energy (MJ/kg)**	16.12	16.12	16.12	17.00	16.79	16.83
**Crude Protein**	21.34	21.14	20.71	19.73	19.20	18.91
**Calcium**	1.26	1.23	1.22	1.12	1.05	1.05
**Total Phosphorus**	0.65	0.67	0.65	0.59	0.60	0.57
**Aflatoxin B_1_**	0.84	0.57	0.54	1.05	1.14	0.95
**Deoxynivalenol**	427	1771	1509	378	1811	1886
**Zearalenone**	21.6	387	360	18.45	429	484

^1^ AME, apparent metabolizable energy; Premix provided per kilogram of diet: vitamin A (transretinyl acetate), 10,000 IU; vitamin D_3_ (cholecalciferol), 3000 IU; vitamin E (all-rac-α-tocopherol), 30 IU; menadione, 1.3 mg; thiamin, 2.2 mg; riboflavin, 8 mg; nicotinamide, 40 mg; choline chloride, 600 mg; calcium pantothenate, 10 mg; pyridoxine·HCl, 4 mg; biotin, 0.04 mg; folic acid, 1 mg; vitamin B_12_ (cobalamin), 0.013 mg; Fe (from ferrous sulphate), 80 mg; Cu (from copper sulphate), 8.0 mg; Mn (from manganese sulphate),110 mg; Zn (from zinc oxide), 60 mg; I (from calcium iodate), 1.1 mg; Se (from sodium selenite),0.3 mg. Analyzed values based on analysis of triplicate samples of diets. ^2^ Control group, Mycotoxins group, and MPal group, broiler ckickens were given the basal diet, and the *Fusarium* mycotoxin-contaminated diets supplemented with 0 and 1 g/kg MPal, respectively.

**Table 7:** Sequences for real-time PCR primers.

Genes ^1^	Gene Bank ID	Primer Sequence (5′-3′)	Length
MUC2	XM_001234581.3	F: AGGAATGGGCTGCAAGAGAC	77
		R: GTGACATCAGGGCACACAGA	
ZO-1	XM_413773.4	F: TGTAGCCACAGCAAGAGGTG	159
		R: CTGGAATGGCTCCTTGTGGT	
OCLN	NM_205128.1	F: CCGTAACCCCGAGTTGGAT	214
		R: CCGTAACCCCGAGTTGGAT	
CLDN2	NM_001277622.1	F: CCTGCTCACCCTCATTGGAG	145
		R: GCTGAACTCACTCTTGGGCT	
CLDN3	NM_204202.1	F: CCCGTCCCGTTGTTGTTTTG	126
		R: CCCCTTCAACCTTCCCGAAA	
β-actin	NM 205518.1	F: TTGGTTTGTCAAGCAAGCGG	100
		R: CCCCCACATACTGGCACTTT	

^1^ MUC2, mucin 2; ZO-1, zonula occludens-1; OCLN, occludin; CLDN2, claudin-2; CLDN3, claudin-3.

## References

[1] Kabak B., Dobson A.D.W., Var I. (2006). Strategies to prevent mycotoxin contamination of food and animal feed: A review. Crit. Rev. Food Sci. Nutr..

[2] Escrivá L., Font G., Manyes L. (2015). In vivo toxicity studies of *Fusarium* mycotoxins in the last decade: A review. Food Chem. Toxicol..

[3] Döll S., Dänicke S. (2011). The *Fusarium* toxins deoxynivalenol (DON) and zearalenone (ZON) in animal feeding. Prev. Vet. Med..

[4] Ren Z., Deng H., Deng Y., Liang Z., Deng J., Zuo Z., Hu Y., Shen L., Yu S., Cao S. (2017). Combined effects of deoxynivalenol and zearalenone on oxidative injury and apoptosis in porcine splenic lymphocytes in vitro. Exp. Toxicol. Pathol..

[5] Zhu L., Yuan H., Guo C., Lu Y., Deng S., Yang Y., Wei Q., Wen L., He Z. (2011). Zearalenone induces apoptosis and necrosis in porcine granulosa cells via a caspase-3- and caspase-9-dependent mitochondrial signaling pathway. J. Cell. Physiol..

[6] Gutleb A.C., Morrison E., Murk A.J. (2002). Cytotoxicity assays for mycotoxins produced by *Fusarium* strains: A review. Environ. Toxicol. Pharmacol..

[7] Chen S.S., Li Y.H., Lin M.F. (2017). Chronic exposure to the *Fusarium* mycotoxin deoxynivalenol: Impact on performance, immune organ, and intestinal integrity of slow-growing chickens. Toxins.

[8] Reddy E.K., Song J., Lee H.J., Kim M., Kim D.W., Jung J.H., Kim B., Lee Y., Yu D., Kim D.W. (2018). Effects of high levels of deoxynivalenol and zearalenone on growth performance, and hematological and immunological parameters in pigs. Toxins.

[9] Borutova R., Faix S., Placha I., Gresakova L., Cobanova K., Leng L. (2008). Effects of deoxynivalenol and zearalenone on oxidative stress and blood phagocytic activity in broilers. Arch. Anim. Nutr..

[10] Shi B., Su Y., Chang S., Sun Y., Meng X., Shan A. (2017). Vitamin C protects piglet liver against zearalenone-induced oxidative stress by modulating expression of nuclear receptors PXR and CAR and their target genes. Food Funct..

[11] Yang X., Li L., Duan Y., Yang X. (2017). Antioxidant activity of *Lactobacillus plantarum* JM113 in vitro and its protective effect on broiler chickens challenged with deoxynivalenol. J. Anim. Sci..

[12] Zhang Y., Gao R., Liu M., Shi B., Shan A., Cheng B. (2015). Use of modified halloysite nanotubes in the feed reduces the toxic effects of zearalenone on sow reproduction and piglet development. Theriogenology.

[13] Chen F., Ma Y., Xue C., Ma J., Xie Q., Wang G., Bi Y., Cao Y. (2008). The combination of deoxynivalenol and zearalenone at permitted feed concentrations causes serious physiological effects in young pigs. J. Vet. Sci..

[14] Wang J.P., Chi F., Kim I.H. (2012). Effects of montmorillonite clay on growth performance, nutrient digestibility, vulva size, faecal microflora, and oxidative stress in weaning gilts challenged with zearalenone. Anim. Feed Sci. Technol..

[15] Martens E.C., Neumann M., Desai M.S. (2018). Interactions of commensal and pathogenic microorganisms with the intestinal mucosal barrier. Nat. Rev. Microbiol..

[16] Jin L., Wang W., Degroote J., Van Noten N., Yan H., Majdeddin M., Van Poucke M., Peelman L., Goderis A., Van De Mierop K. (2017). Mycotoxin binder improves growth rate in piglets associated with reduction of toll-like receptor-4 and increase of tight junction protein gene expression in gut mucosa. J. Anim. Sci. Biotechnol..

[17] Liu M., Zhu D., Guo T., Zhang Y., Shi B., Shan A., Chen Z. (2017). Toxicity of zearalenone on the intestines of pregnant sows and their offspring and alleviation with modified halloysite nanotubes.. J. Sci. Food Agric..

[18] Antonissen G., Van Immerseel F., Pasmans F., Ducatelle R., Janssens G.P.J., De Baere S., Mountzouris K.C., Su S., Wong E.A., De Meulenaer B. (2015). Mycotoxins deoxynivalenol and fumonisins alter the extrinsic component of intestinal barrier in broiler chickens. J. Agric. Food Chem..

[19] Przybylska-Gornowicz B., Tarasiuk M., Lewczuk B., Prusik M., Ziółkowska N., Zielonka Ł., Gajęcki M., Gajęcka M. (2015). The effects of low doses of two *Fusarium* toxins, zearalenone and deoxynivalenol, on the pig jejunum. A light and electron microscopic study. Toxins.

[20] Alizadeh A., Braber S., Akbari P., Garssen J., Fink-Gremmels J. (2015). Deoxynivalenol impairs weight gain and affects markers of gut health after low-dose, short-term exposure of growing pigs. Toxins.

[21] Vila-Donat P., Marín S., Sanchis V., Ramos A.J. (2018). A review of the mycotoxin adsorbing agents, with an emphasis on their multi-binding capacity, for animal feed decontamination. Food Chem. Toxicol..

[22] Di Gregorio M.C., Neeff D.V.D., Jager A.V., Corassin C.H., Carão Á.C.D.P., Albuquerque R.D., Azevedo A.C.D., Oliveira C.A.F. (2014). Mineral adsorbents for prevention of mycotoxins in animal feeds. Toxin Rev..

[23] Avantaggiato G., Solfrizzo M., Visconti A. (2005). Recent advances on the use of adsorbent materials for detoxification of *Fusarium* mycotoxins. Food Addit. Contam..

[24] Kong C., Shin S.Y., Kim B.G. (2014). Evaluation of mycotoxin sequestering agents for aflatoxin and deoxynivalenol: An in vitro approach. Springerplus.

[25] Yiannikouris A., Kettunen H., Apajalahti J., Pennala E., Moran C.A. (2013). Comparison of the sequestering properties of yeast cell wall extract and hydrated sodium calcium aluminosilicate in three in vitro models accounting for the animal physiological bioavailability of zearalenone. Food Addit. Contam. A.

[26] Döll S., Dänicke S., Valenta H., Flachowsky G. (2004). In vitro studies on the evaluation of mycotoxin detoxifying agents for their efficacy on deoxynivalenol and zearalenone. Arch. Anim. Nutr..

[27] Zhang Y., Gao R., Liu M., Yan C., Shan A. (2014). Adsorption of modified halloysite nanotubes in vitro and the protective effect in rats exposed to zearalenone. Arch. Anim. Nutr..

[28] Li Y., Tian G., Dong G., Bai S., Han X., Liang J., Meng J., Zhang H. (2018). Research progress on the raw and modified montmorillonites as adsorbents for mycotoxins: A review. Appl. Clay Sci..

[29] Jiang S.Z., Yang Z.B., Yang W.R., Wang S.J., Liu F.X., Johnston L.A., Chi F., Wang Y. (2012). Effect of purified zearalenone with or without modified montmorillonite on nutrient availability, genital organs and serum hormones in post-weaning piglets. Livest. Sci..

[30] Döll S., Gericke S., Dänicke S., Raila J., Ueberschär K.H., Valenta H., Schnurrbusch U., Schweigert F.J., Flachowsky G. (2005). The efficacy of a modified aluminosilicate as a detoxifying agent in *Fusarium* toxin contaminated maize containing diets for piglets. J. Anim. Physiol. Anim. Nutr..

[31] Zhou H. (2016). Mixture of palygorskite and montmorillonite (Paly-Mont) and its adsorptive application for mycotoxins. Appl. Clay Sci..

[32] Schell T.C., Lindemann M.D., Kornegay E.T., Blodgett D.J., Doerr J.A. (1993). Effectiveness of different types of clay for reducing the detrimental effects of aflatoxin-contaminated diets on performance and serum profiles of weanling pigs. J. Anim. Sci..

[33] Lindemann M.D., Blodgett D.J., Harper A.F., Kornegay E.T., Doerr J.A. (1997). Appraisal of the value of selected clays and minerals in diets with and without aflatoxin-contaminated maize fed to young pigs. J. Anim. Feed Sci..

[34] Chen Y.P., Cheng Y.F., Li X.H., Zhang H., Yang W.L., Wen C., Zhou Y.M. (2016). Dietary palygorskite supplementation improves immunity, oxidative status, intestinal integrity, and barrier function of broilers at early age. Anim. Feed Sci. Technol..

[35] Zhang J., Lv Y., Tang C., Wang X. (2013). Effects of dietary supplementation with palygorskite on intestinal integrity in weaned piglets. Appl. Clay Sci..

[36] Qiao L.H., Chen Y.P., Wen C., Zhou Y.M. (2015). Effects of natural and heat modified palygorskite supplementation on the laying performance, egg quality, intestinal morphology, digestive enzyme activity and pancreatic enzyme mRNA expression of laying hens. Appl. Clay Sci..

[37] Su Y., Chen Y.P., Chen L.J., Xu Q., Kang Y.R., Wang W.B., Wang A.Q., Wen C., Zhou Y.M. (2018). Effects of different levels of modified palygorskite supplementation on the growth performance, immunity, oxidative status and intestinal integrity and barrier function of broilers. J. Anim. Physiol. Anim. Nutr..

[38] Su Y., Chen Y.P., Cheng Y.F., Wen C., Zhou Y.M. (2018). Effects of modified palygorskite supplementation on egg quality and mineral element content, and intestinal integrity and barrier function of laying hens. Biol. Trace Elem. Res..

[39] Chen L.J. (2017). Application of Attapulgite Adsorbent for Zearalenone in Feed of Broilers. Master’s Thesis.

[40] Xu Q. (2018). A Study of the Protective Effects of Modified Palygorskite on Broilers Ingesting Diets Contaminated with Zearalenone. Master’s Thesis.

[41] Li Z., Yang Z.B., Yang W.R., Wang S.J., Jiang S.Z., Wu Y.B. (2012). Effects of feed-borne *Fusarium* mycotoxins with or without yeast cell wall adsorbent on organ weight, serum biochemistry, and immunological parameters of broiler chickens. Poult. Sci..

[42] Wan M.L.Y., Turner P.C., Allen K.J., El-Nezami H. (2016). *Lactobacillus rhamnosus* GG modulates intestinal mucosal barrier and inflammation in mice following combined dietary exposure to deoxynivalenol and zearalenone. J. Funct. Foods.

[43] Swamy H.V.L.N., Smith T.K., Karrow N.A., Boermans H.J. (2004). Effects of feeding blends of grains naturally contaminated with *Fusarium* mycotoxins on growth and immunological parameters of broiler chickens. Poult. Sci..

[44] Girish C.K., Smith T.K. (2008). Effects of feeding blends of grains naturally contaminated with *Fusarium* mycotoxins on small intestinal morphology of turkeys. Poult. Sci..

[45] Yegani M., Smith T.K., Leeson S., Boermans H.J. (2006). Effects of feeding grains naturally contaminated with *Fusarium* mycotoxins on performance and metabolism of broiler breeders. Poult. Sci..

[46] Kubena L.F., Huff W.E., Harvey R.B., Corrier D.E., Phillips T.D., Creger C.R. (1988). Influence of ochratoxin A and deoxynivalenol on growing broiler chicks. Poult. Sci..

[47] Corthesy B. (2013). Multi-faceted functions of secretory IgA at mucosal surfaces. Front. Immunol..

[48] Yin S., Meng Q., Zhang B., Shi B., Shan A., Li Z. (2015). Alleviation of zearalenone toxicity by modified halloysite nanotubes in the immune response of swine. Food Addit. Contam. Part A.

[49] Qu X.Y., Chen J.F., He C.Q., Chi F., Johnston S.L. (2018). Effects of modified montmorillonite adsorbent on performance, egg quality, serum biochemistry, oxidation status, and immune response of laying hens in late production, *Livest*. Sci..

[50] Weaver C.A., See T.M., Hansen A.J., Kim B.Y., De Souza L.A., Middleton F.T., Kim W.S. (2013). The use of feed additives to reduce the effects of aflatoxin and deoxynivalenol on pig growth, organ health and immune status during chronic exposure. Toxins.

[51] Ayala A., Muñoz M.F., Argüelles S. (2014). Lipid peroxidation: Production, metabolism and signaling mechanisms of malondialdehyde and 4-hydroxy-2-nonenal. Oxid. Med. Cell. Longev..

[52] Zelko I.N., Mariani T.J., Folz R.J. (2002). Superoxide dismutase multigene family: A comparison of the CuZn-SOD (SOD1), Mn-SOD (SOD2), and EC-SOD (SOD3) gene structures, evolution, and expression. Free Radic. Biol. Med..

[53] Jiang S.Z., Yang Z.B., Yang W.R., Wang S.J., Wang Y., Broomhead J., Johnston S.L., Chi F. (2011). Effect on hepatonephric organs, serum metabolites and oxidative stress in post-weaning piglets fed purified zearalenone-contaminated diets with or without Calibrin-Z.. J. Anim. Physiol. Anim. Nutr..

[54] Liu M., Gao R., Meng Q., Zhang Y., Bi C., Shan A. (2014). Toxic effects of maternal zearalenone exposure on intestinal oxidative stress, barrier function, immunological and morphological changes in rats. PLoS ONE.

[55] Rocha O., Ansari K., Doohan F.M. (2005). Effects of trichothecene mycotoxins on eukaryotic cells: A review. Food Addit. Contam..

[56] Wolvekamp M.C.J., de Bruin R.W.F. (1994). Diamine oxidase: An overview of historical, biochemical and functional aspects. Dig. Dis..

[57] Tsunooka N., Maeyama K., Nakagawa H., Doi T., Horiuchi A., Miyauchi K., Watanabe Y., Imagawa H., Kawachi K. (2006). Localization and changes of diamine oxidase during cardiopulmonary bypass in rabbits. J. Surg. Res..

[58] Wu M., Xiao H., Ren W., Yin J., Tan B., Liu G., Li L., Nyachoti C.M., Xiong X., Wu G. (2014). Therapeutic effects of glutamic acid in piglets challenged with deoxynivalenol. PLoS ONE.

[59] Hutsko S.L., Meizlisch K., Wick M., Lilburn M.S. (2016). Early intestinal development and mucin transcription in the young poult with probiotic and mannan oligosaccharide prebiotic supplementation. Poult. Sci..

[60] Lerner A., Matthias T. (2015). Changes in intestinal tight junction permeability associated with industrial food additives explain the rising incidence of autoimmune disease. Autoimmun. Rev..

[61] Goossens J., Pasmans F., Verbrugghe E., Vandenbroucke V., De Baere S., Meyer E., Haesebrouck F., De Backer P., Croubels S. (2012). Porcine intestinal epithelial barrier disruption by the *Fusarium* mycotoxins deoxynivalenol and T-2 toxin promotes transepithelial passage of doxycycline and paromomycin. BMC Vet. Res..

[62] Liao P., Liao M., Li L., Tan B., Yin Y. (2017). Effect of deoxynivalenol on apoptosis, barrier function, and expression levels of genes involved in nutrient transport, mitochondrial biogenesis and function in IPEC-J2 cells. Toxicol. Res..

[63] Akbari P., Braber S., Alizadeh A., Verheijden K.A.T., Schoterman M.H.C., Kraneveld A.D., Garssen J., Fink-Gremmels J. (2015). Galacto-oligosaccharides protect the intestinal barrier by maintaining the tight junction network and modulating the inflammatory responses after a challenge with the mycotoxin deoxynivalenol in human Caco-2 cell monolayers and B6C3F1 mice. J. Nutr..

[64] NRC (1994). Nutrient Requirements of Poultry.

[65] Cheng Y.F., Chen Y.P., Wen C., Wang W.B., Wang A.Q., Zhou Y.M. (2018). Evaluation of dietary palygorskite supplementation on growth performance, mineral accumulations, antioxidant capacities, and meat quality of broilers fed lead-contaminated diet. Biol. Trace Elem. Res..

[66] Hosoda N., Nishi M., Nakagawa M., Hiramatsu Y., Hioki K., Yamamoto M. (1989). Structural and functional alterations in the gut of parenterally or enterally fed rats. J. Surg. Res..

[67] Placer Z.A., Cushman L.L., Johnson B.C. (1966). Estimation of product of lipid peroxidation (malonyl dialdehyde) in biochemical systems. Anal. Biochem..

[68] Sun Y., Oberley L.W., Li Y. (1988). A simple method for clinical assay of superoxide dismutase. Clin. Chem..

[69] Hu C.H., Qian Z.C., Song J., Luan Z.S., Zuo A.Y. (2013). Effects of zinc oxide-montmorillonite hybrid on growth performance, intestinal structure, and function of broiler chicken. Poult. Sci..

[70] Chen Y.P., Zhang H., Cheng Y.F., Li Y., Wen C., Zhou Y.M. (2018). Dietary l-threonine supplementation attenuates lipopolysaccharide-induced inflammatory responses and intestinal barrier damage of broiler chickens at an early age. Br. J. Nutr..

[71] Livak K.J., Schmittgen T.D. (2001). Analysis of relative gene expression data using real-time quantitative PCR and the 2^−ΔΔCT^ method. Methods.

